# Impact of the first response unit on prehospital on-scene time among paramedic-suspected stroke patients: a retrospective before–after cohort study in Finland

**DOI:** 10.1186/s13049-023-01089-7

**Published:** 2023-06-13

**Authors:** Verna K.E. Vaajanen, Pauli E.T. Vuorinen, Piritta A. Setälä, Reija Autio, Sanna E. Hoppu

**Affiliations:** 1 Faculty of Medicine and Health Technology, Tampere University, Tampere, Finland; 2 Emergency Medical Services, Centre for Prehospital Emergency Care, Department of Emergency, Anaesthesia and Pain Medicine, Tampere University Hospital, Tampere, Finland; 3 Faculty of Social Sciences, Tampere University, Tampere, Finland

**Keywords:** First response unit, On-scene time, Stroke, Emergency medical services

## Abstract

**Background:**

Revascularization of an occluded artery by either thrombolysis or mechanical thrombectomy is a time-critical intervention in ischaemic stroke. Each link in the stroke chain of survival should minimize the delay to definitive treatment in every possible way. In this study, we investigated the effect of routine dispatch of a first response unit (FRU) on prehospital on-scene time (OST) on stroke missions.

**Methods:**

Medical dispatch of FRU together with an emergency medical service (EMS) ambulance was a routine strategy in the Tampere University Hospital area before 3 October 2018, after which the FRU has only been dispatched to medical emergencies on the decision of an EMS field commander. This study presents a retrospective before–after analysis of 2,228 paramedic-suspected strokes transported by EMSs to Tampere University Hospital. We collected data from EMS medical records from April 2016 to March 2021, and used statistical tests and binary logistic regression to detect the associations between the variables and the shorter and longer half of OSTs.

**Results:**

The median OST of stroke missions was 19 min, IQR [14–25] min. The OST decreased when the routine use of the FRU was discontinued (19 [14–26] min vs. 18 [13–24] min, *p* < 0.001). The median OST with the FRU being the first at the scene (*n* = 256, 11%) was shorter than in cases where the FRU arrived after the ambulance (16 [12–22] min vs. 19 [15–25] min, *p* < 0.001). The OST with a stroke dispatch code was shorter than with non-stroke dispatches (18 [13–23] min vs. 22 [15–30] min, *p* < 0.001). The OST for thrombectomy candidates was shorter than that for thrombolysis candidates (18 [13–23] min vs. 19 [14–25], *p* = 0.01). The shorter half of OSTs were associated with the FRU arriving first at the scene, stroke dispatch code, thrombectomy transportation and urban location.

**Conclusion:**

The routine dispatch of the FRU to stroke missions did not decrease the OST unless the FRU was first to arrive at the scene. In addition, a correct stroke identification in the dispatch centre and thrombectomy candidate status decreased the OST.

## Background

Revascularization therapy for ischaemic stroke by either pharmacological thrombolysis or mechanical thrombectomy has been the treatment of choice for the last 25 years [1]. Resolution of stroke symptoms and the return to everyday activities are only possible when revascularization occurs before irreversible neural destruction in the brain. The crucial factors in recovery from an ischaemic stroke may be described as a timeline in the stroke chain of survival: time from the onset of symptoms to call, call to dispatch, dispatch to the scene, on-scene time (OST), transport time, door-to-treatment time and post-stroke rehabilitation [2]. The possibilities of reducing the time from onset to call are dependent on public awareness and on educational campaigns on ischaemic stroke [3]. The time from the beginning of the emergency call to the dispatch of the emergency medical services (EMSs) is approximately 3 min [4]. In a wider perspective, speeding up the dispatch protocol does not significantly shorten the onset-to-revascularization time. However, the accuracy of the dispatch code does affect the timely treatment on the scene. Paramedics use routine stroke scores and recognize a stroke better when they are dispatched with a suspected stroke dispatch code [5]. One of the most important factors for the paramedics in reducing the time to definitive treatment is correct stroke recognition with prenotification to the receiving hospital [6]. It has been speculated that the on-scene time in high workload call-outs and in difficult evacuations can be reduced with the help of a first response unit (FRU) and by performing only vital manoeuvres on the scene. Until October 2018, the FRU was routinely dispatched to all suspected strokes in the Tampere University Hospital area, which consists of urban, semi-urban and rural areas. Subsequently, all routine FRU dispatch was discontinued in the Tampere University Hospital area. This happened after a Finnish study showed no reduction of on-scene time with the FRU in urban areas [7].

In this retrospective before–after cohort study, we aimed to investigate how the FRU dispatch affected on-scene time among paramedic-suspected stroke patients and thus whether it was an appropriate decision in practice to restructure the system by discontinuing the routine use of the FRU in urban and rural areas according to an earlier study of urban areas. Our hypothesis was that the routine use of the FRU decreases OST as a result of the FRU having prepared the stroke patient ready for ambulance transportation, thus accelerating the operation especially in more rural areas. In addition, we wanted to look at some of the factors that may be associated with a shorter on-scene time, and which have not previously been investigated, namely dispatch code, sequence of arrival on the scene, patient location, timing, and categorization into thrombolysis and thrombectomy candidates.

## Methods

### Aim of the study

Our aim was to investigate the impact of the first response unit on on-scene time among stroke patients and the variables associated with shorter on-scene time.

### Study design

This cohort study is a retrospective before–after analysis of the FRU’s impact on OST among acute paramedic-suspected stroke patients.

### Setting

The Tampere University Hospital area has a population of 530,000 people and a surface area of 16 000 km^2^. The area consists of the urban city of Tampere, semi-urban municipalities around the city, and large surrounding rural areas of dispersed settlement. Local EMSs transport all paramedic-suspected strokes to Tampere University Hospital’s emergency department, which serves as a comprehensive stroke centre (CSC) for a population of 1,000,000 people. The CSC has 24/7 procedure readiness.

### Emergency dispatch

The Finnish emergency response centre agency receives all emergency calls dialled to the number 112 and directs them to the appropriate authorities and gives guidance to the person calling. The emergency dispatcher evaluates the situation and determines a dispatch code and a classification of urgency. The stroke dispatch code is used when the caller suspects a stroke or mentions facial asymmetry, difficulty in speaking or weakness or numbness in a pair of limbs. During the period of the present study, if stroke symptoms had emerged within 6 h or if the patient woke up with them, the EMS responded with lights and sirens. As a result of the thrombectomy candidate needing a different kind of team at the receiving hospital compared to the thrombolysis candidate, a separate transportation code for thrombectomy candidates was introduced in May 2017. However, this did not change the on-site treatment of the patient.

### Emergency medical services

The ambulance crew dispatched to suspected stroke missions consists of two advanced life support (ALS) level nurse paramedics or one ALS nurse paramedic with an emergency medical technician or firefighters. Qualification as a nurse paramedic involves a four-year degree from a university of applied sciences for health care. Previously, nurse paramedics were taught to recognize stroke patients by using the face-arm-speech triad, but with the evolution of interventional radiology in the treatment of large vessel occlusions (LVOs), intensive training was implemented for paramedics. Since 2017, they have screened patients as candidates for thrombolytic therapy and mechanical thrombectomy by using the Finnish Prehospital Stroke Scale [8].

Finnish regulations define the FRU as any EMS or rescue unit most likely to reach the patient first. The FRU unit must have at least two first responders with certification to provide first aid. In this study all the FRUs were either professional or voluntary fire engine supports. The purpose of the FRU is to speed up the onset of life-saving treatment or to provide any aid to EMS dispatches with a high workload. The FRU may be dispatched in addition to an ambulance, but it never transports the patient and seldom gives any medication or intravenous infusions. The FRUs serve especially semi-urban and rural areas in Finland where distances to the nearest hospital are long, and the nearest EMS unit might be preoccupied with another mission. After the protocol change the unit was dispatched only if the EMS field commander, an operative chief of the EMS, considered that it would reach the patient significantly faster or that the situation otherwise needed extra help at the scene.

### Subjects

We collected data on the EMS dispatches with paramedic-suspected stroke transports with timeline data. This covered 2.5 years during the routine use of the FRU (1 April 2016 to 3 October 2018) and 2.5 years after discontinuing the routine use of the FRU (4 October 2018 to 31 March 2021). The data contained dates, addresses, dispatch and transport codes, classes of urgency, prehospital timeline, and information about alerting the FRU and its presence at the scene, but no individual patient data. Timeline data were collected from paramedics using the manual or electronic timestamps routinely recorded during the mission. The timestamps were collected from the real-time and computer assisted clocks of the national dispatch system and from ambulance computers. Paramedics used manual registration with the mobile electronic patient reporting computer for on-scene arrival and transport timestamps. These data were saved in the Codea database (Codea Ltd, Tampere, Finland) of the Tampere University Hospital area.

### Data

OSTs were calculated using prehospital timestamps. We categorized the usage of the FRU into “during routine use” and “after routine use was discontinued”, the order of arrival at the scene into “FRU first” and “ambulance first”, dispatch codes into “stroke” and “non-stroke” and transportation urgencies into “thrombectomy” and “thrombolysis”. In our model, we categorized the towns within the area based on “city” for Tampere, “surrounding municipalities” for Kangasala, Lempäälä, Nokia, Orivesi, Pirkkala, Vesilahti and Ylöjärvi, and the rest as “dispersed settlement”. Timing was categorized into “office hours”, 7.30 AM–4 PM, and “on-call”, which included weekends, holidays, evenings, and nights (Table 1).

**Table 1:** Number of cases per each group. FRU, first response unit

Group	All cases (%) *N* = 2206	Shortest half (%) *N* = 1183	Longest half (%) *N* = 1023
Use of the FRU			
During the routine use of the FRU	1071 (48.5)	545 (46.1)	526 (51.4)
After the routine use of the FRU	1135 (51.5)	638 (53.9)	497 (48.6)
First at the scene			
Ambulance first at the scene	301 (13.6)	158 (13.4)	143 (14.0)
The FRU first at the scene	252 (11.4)	166 (14.0)	86 (8.4)
Dispatch code			
Non-stroke	662 (30.0)	268 (22.7)	394 (38.5)
Stroke	1544 (70.0)	915 (77.3)	629 (61.5)
Transportation urgency			
Thrombolysis candidate	1838 (83.3)	969 (81.9)	869 (84.9)
Thrombectomy candidate	368 (16.7)	214 (18.1)	154 (15.1)
Location			
Dispersed settlement	837 (37.9)	400 (33.8)	437 (42.7)
City (Tampere)	772 (35.0)	426 (36.0)	346 (33.8)
Surrounding municipalities	597 (27.1)	357 (30.2)	240 (23.5)
Timing			
On-call	1297 (58.8)	670 (56.6)	627 (61.3)
Office hours	909 (41.2)	513 (43.4)	396 (38.7)

### Statistical analyses

We used the Chi-squared test for categorical variables and the Mann–Whitney *U* test to compare numerical variables. Binary logistic regression models were used to detect the associations between the exposures, i.e., the use of the FRU, first at the scene, dispatch code, transportation urgency, location, and timing, and outcome of the shorter OST. First, univariate models were created separately for each variable. Next, all the variables were added into the same binary logistic model that we used to compute the adjusted odds ratios (ORs) between the variables and the outcome (Table 2). Unadjusted and adjusted ORs with 95% confidence intervals (CIs) were computed. All statistical analyses were carried out using IBM SPSS Statistics for Macintosh (version 28.0.0). The results with a p-value of < 0.05 were considered statistically significant.

**Table 2:** Factors associated with shorter on-scene time. FRU, first response unit

	Univariate model		Adjusted model	
	**OR [95% CI]**	***p*** **-value**	**OR [95% CI]**	***p*** **-value**
Use of the FRU				
During the routine use of the FRU	1.0		1.0	
After the routine use of the FRU	1.2 [1.0–1.5]	0.01	0.6 [0.3–1.2]	0.1
First at the scene				
Ambulance first at the scene	1.0		1.0	
The FRU first at the scene	1.7 [1.2–2.5]	0.002	1.7 [1.2–2.5]	0.004
Dispatch code				
Non-stroke	1.0		1.0	
Stroke	2.1 [1.8–2.6]	< 0.001	4.3 [2.1–9.0]	< 0.001
Transportation urgency				
Thrombolysis candidate	1.0		1.0	
Thrombectomy candidate	1.2 [1.0–1.6]	0.06	2.4 [1.2–4.7]	0.009
Location				
Dispersed settlement	1.0		1.0	
City (Tampere)	1.3 [1.1–1.6]	0.003	1.9 [1.3–3.0]	0.003
Surrounding municipalities	1.6 [1.3–2.0]	< 0.001	1.9 [1.2–3.0]	0.008
Timing				
On-call	1.0		1.0	
Office hours	1.2 [1.0–1.4]	0.03	1.4 [1.0–2.0]	0.08

## Results

### Sample

**Fig. 1:**
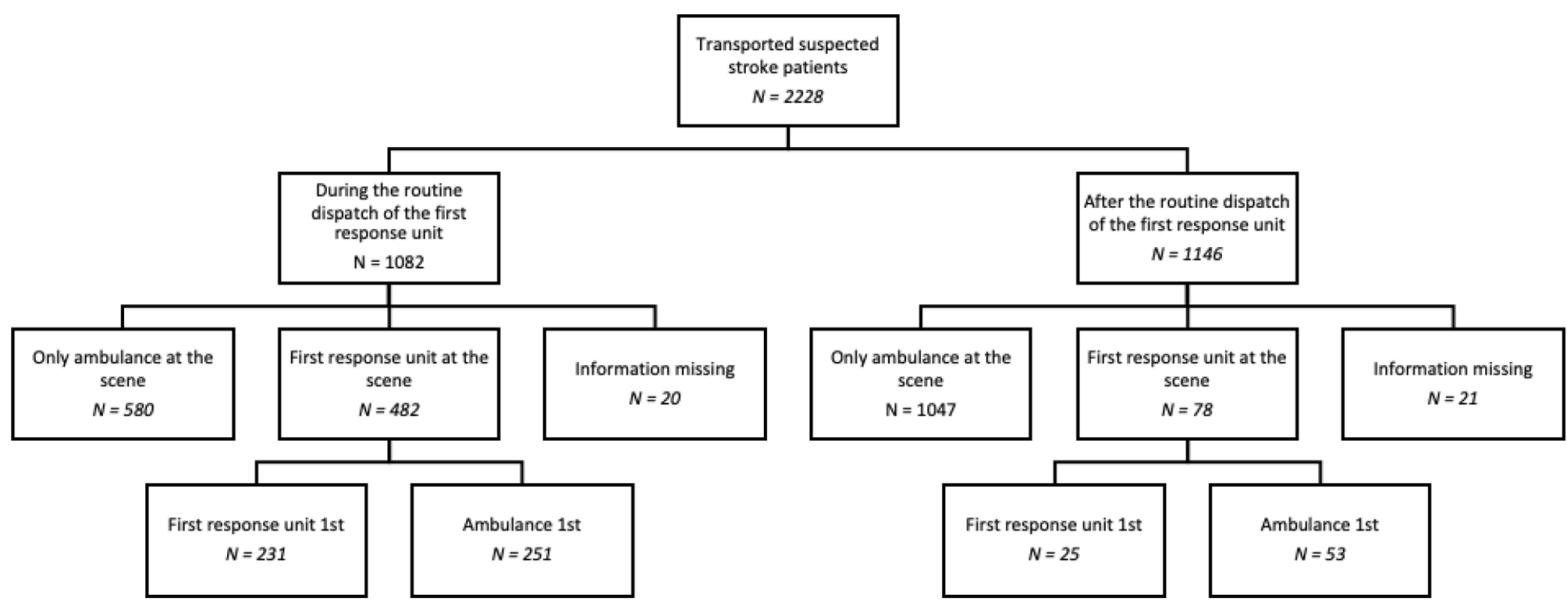
Patient flow

During the study period, EMSs transported a total of 2,228 paramedic-suspected stroke patients to Tampere University Hospital. Of these transportations, 1,562 (70%) were dispatched with a suspected stroke code. There were 1,082 transportations during the routine use of the FRU and 1,146 after the routine use of the FRU was discontinued. The patient flow is presented in Fig. 1. During the time the FRU was routinely used, the FRU was at the scene in 482 missions and arrived first in 231 (48%). After routine use was discontinued, the FRU was at the scene in 78 missions and arrived first in 25 (32%). The stroke dispatches were urgent in 97% of the cases, while only 72% of non-stroke dispatches were urgent (*p* < 0.001). After a separate transportation code for thrombectomy candidates was introduced, the dispatcher used a suspected stroke dispatch code for 217 (59%) patients out of 365 transportations for thrombectomy candidates. For thrombolysis candidates, 1,055 (73%) of 1,452 transportations were dispatched with a suspected stroke dispatch code (*p* < 0.001). From the total of 2,228 stroke transportations, there were 22 (1.0%) cases with missing OST information. There were 41 (1.8%) stroke transportations with missing information on the presence of the FRU at the scene.

### On-scene times

**Fig. 2:**
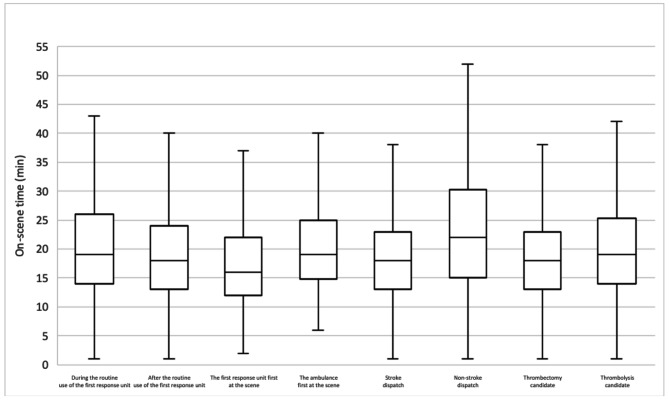
Box plot of stroke transportations on-scene times (min). Whiskers indicate the minimum and the maximum. Outliers are excluded from the figure

The median and quartiles of the OST of 2,206 paramedic-suspected stroke transportations were 19 [14–25] min. During the time of the routine use of the FRU, the OST was 19 [14–26] min, and after discontinuing the FRU, the OST was 18 [13–24] min (*p* < 0.001). In the entire data, whenever the FRU was present at the scene, the OST was 18 [14–23] min compared to that of the ambulance crew working alone (19 [14–26] min, *p* = 0.05). In addition, when the FRU arrived first at the scene, the OST decreased by 3 min compared to the FRU arriving after the ambulance (16 min [12–22] vs. 19 min [15–25] min, *p* < 0.001). Correct stroke identification by the emergency dispatcher decreased the OST by 4 min (18 [13–23] min vs. 22 [15–30] min, *p* < 0.001). Thrombectomy candidates had a shorter OST than thrombolysis candidates (18 [13–23] min vs. 19 [14–25] min, *p* = 0.01). The distributions of the OSTs are presented in Fig. 2.

To detect the variables associated with short OSTs, the data were dichotomized into the shortest and longest half according to the median of all OSTs. Binary logistic regression analysis was run to detect the associations between the shorter and the longer half of OSTs. The factors associated with the shorter half of OSTs were as follows: stroke dispatch code (OR 4.3, 95% CI 2.1–9.0, *p* < 0.001), thrombectomy transportation (OR 2.4, 95% CI 1.2–4.7, *p* = 0.009), patient location, i.e., whether in the city (OR 1.9, 95% CI 1.3–3.0, *p* = 0.003) or in one of the surrounding municipalities (OR 1.9, 95% CI 1.2–3.0, *p* = 0.008) and the FRU arriving first at the scene (OR 1.7, 95% CI 1.2–2.5, *p* = 0.004) (Table 2).

## Discussion

During the time-period when the routine dispatch of the FRU was in use, the OST was higher. The OST decreased only if the FRU arrived at the scene before an ambulance. Thus, it was appropriate to discontinue the routine and dispatch the FRU only on the decision of the field commander. The factors decreasing the OST were the correct dispatch code, thrombectomy transportation and the urban location of the patient.

In our study, the median OST of all missions was 19 min and the shortest median was 16 min in missions with the FRU arriving first at the scene. The Heart and Stroke Foundation of Canada recommends a median OST of 20 min or less when treating a stroke patient on the scene [9]. The American Stroke Association recommends an OST of less than 15 min [10]. In earlier studies, median OSTs varied between 14 and 31.5 min [7, 11–15]. This study agrees with Abbas et al. [15] that OST may be reduced with correct stroke identification. In a small urban study, increasing staffing on the scene did not prove to be time-saving [7]. The OST has been reported to decrease as a result of EMS training programmes [16] as well as by in-hospital ECG recording and by performing intravenous cannulation during transportation [11]. Previous studies have reported an increase in OST related to onboard computed tomography angiography [17], direct endovascular thrombectomy team notification [14] and each 10-year increase in patients’ age [18].

In Abbas et al. [15], the emergency dispatcher’s accuracy in stroke identification was lower than that found in this study (57% vs. 70%). Unlike previous studies, our study compared the differences in identifying thrombectomy and thrombolysis candidates. The proximity of a primary stroke centre, better stroke recognition of thrombectomy candidates and, furthermore, differentiation between thrombolysis and thrombectomy candidates during the emergency call could prove to be valuable [19, 20]. The early addition of a helicopter EMS unit for the dispatch of candidates for revascularization therapies could drastically decrease the time to recanalization [21].

The FRUs in the city of Tampere and in its surroundings are mainly professional firefighters. We have previously shown differences between professional and volunteer FRUs [22], and it is possible that background training explains most of the regional differences in our analysis. Nonetheless, the FRU examines the patient´s condition and its report from the scene gives the paramedics time to plan their working tactics while en route to the destination. The FRU can identify stroke symptoms and forward this information so that the paramedics will focus their tactics on the necessary procedures at the scene. Unfortunately, we were not able to distinguish between a professional and a volunteer FRU attending the scene.

We were surprised that the OST was faster when the patient was a paramedic-suspected thrombectomy candidate. We anticipated that patients with an LVO stroke and hence more severe stroke symptoms would delay the start of transport as they almost always needed to be carried to the ambulance. On the other hand, intensive training to recognize LVO was initiated, and its time-critical nature was emphasized in 2017. Symptoms of an LVO are severe and clear, and actions at the scene are straightforward. It would be intriguing to further explore whether paramedics act differently at the scene depending on whether the patient is a thrombolysis or a thrombectomy candidate.

### Strengths and limitations

This study’s strengths were its large study population and low percentage of missing data, as only 22 (1.0%) of the stroke transportation on-scene times were not available. The limitations were that we did not have any specific data about individual patients´ characteristics, such as weight or information about the final discharge diagnosis. This meant we had no information on how accurately paramedics assessed the need for thrombectomy or thrombolysis. We did not calculate the time needed to arrive at the scene or how much faster a FRU was at the scene than an ambulance. In Finland the annual stroke incidence is approximately 200/100,00 [23] and only a small amount are recanalization candidates. Inevitably false positives – stroke mimics – must have been included in our material due to the compulsory time-sensitive timetable adhere to by paramedics while handling stroke patients. The inclusion of mimics can result from the paucity of patient information available to the emergency dispatcher and EMS, as well as the potentially limited medical expertise of the EMS in differentiate diagnosing, and the lack of sensitivity in pre-hospital scales. Neurological mimics primarily consist of seizures, migraines, and peripheral neuropathies, whereas non-neurological mimics involve cardiovascular infections, mental disorders, and infections. [24] Paramedics do not identify all strokes but, due to the great number of on-scene times in this study, a few missed strokes do not distort the median. In addition, the separate transportation code for thrombectomy candidates and active training of EMS personnel over the years must have decreased OSTs. Furthermore, after discontinuing the routine procedure of dispatching the FRU, the EMS field commander might have initiated the dispatch only after the paramedics had recognized their need for help in evacuating the patient, hence resulting in the later arrival of the FRU on the scene.

## Conclusion

This study reinforces that the routine use of the FRU is not beneficial in stroke missions. It also shows that the OST is shortened by the following factors: the FRU arriving first on the scene, the correct stroke dispatch code, thrombectomy candidate status and an urban location.

## Data Availability

The datasets used and/or analysed during the current study are available from the corresponding author on reasonable request.

## Abbreviations

ALS: Advanced life support
BLS: Basic life support
CSC: Comprehensive stroke centre
EMS: Emergency medical service
FRU: First response unit
LVO: Large vessel occlusion
OR: Odds ratio
OST: On-scene time
95% CI: 95% confidence interval
